# Supplementary material for: Causal interplay between lactose intolerance and gut microbiota: a combined bidirectional Mendelian randomization and in vivo validation study
Source: Front Nutr. 2026 Jun 1;13:1803337. doi: 10.3389/fnut.2026.1803337 (PMC13265576; doi:10.3389/fnut.2026.1803337)
Supplement: Supplementary file 1 [file Data_Sheet_1.zip › supplementary materials/Forward/leave one out cross validation/ebi-a-GCST90027662.finngen_R12_E4_LACTONAS.pdf]

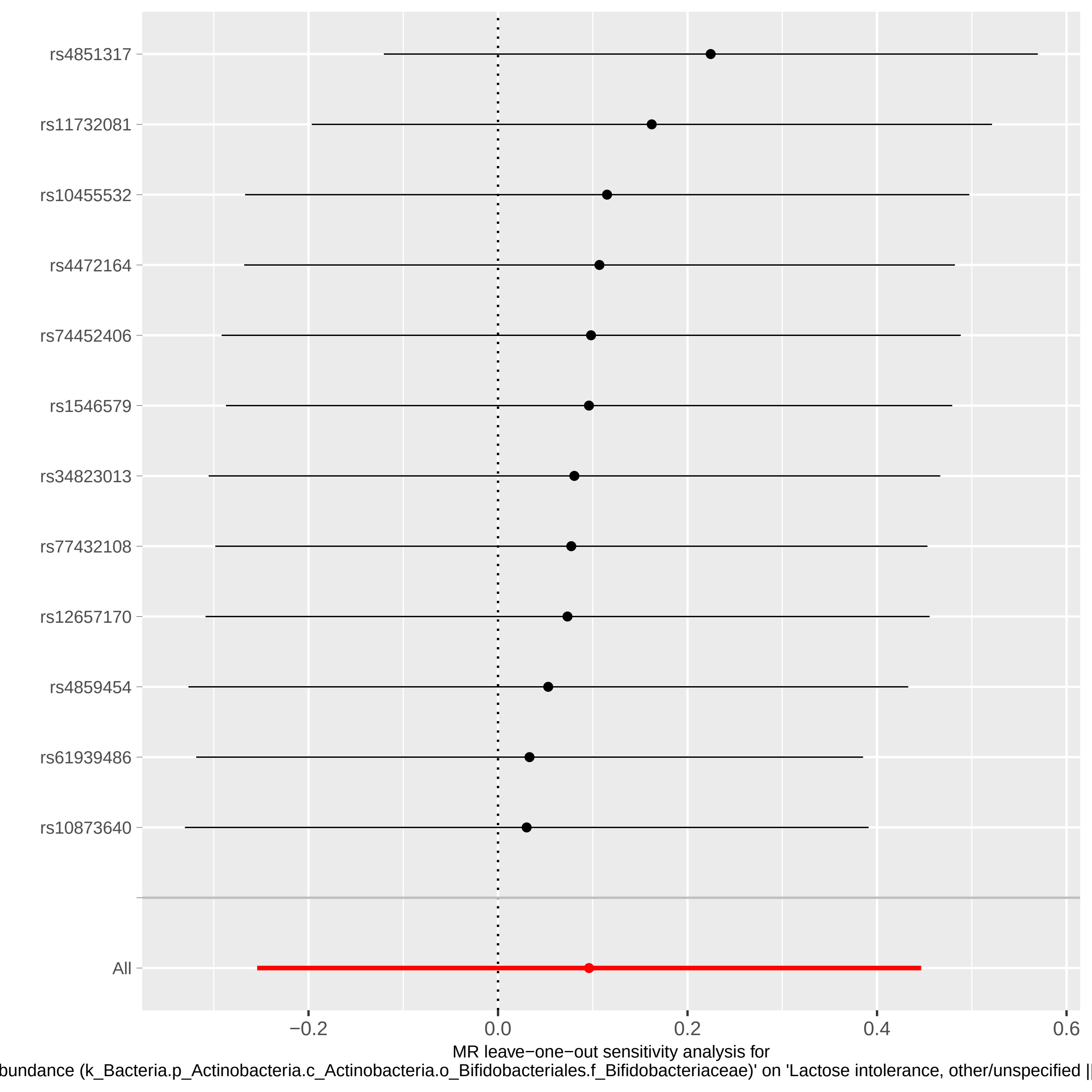

rs4851317
rs11732081
rs10455532
rs4472164
rs74452406
rs1546579
rs34823013
rs77432108
rs12657170
rs4859454
rs61939486
rs10873640
All
−0.2
0.0
0.2
0.4
0.6
MR leave−one−out sensitivity analysis for
bundance (k_Bacteria.p_Actinobacteria.c_Actinobacteria.o_Bifidobacteriales.f_Bifidobacteriaceae)' on 'Lactose intolerance, other/unspecified ||
